# Ocular Surface Microbiota Alterations Following FS‐LASIK and Their Association With Postoperative Dry Eye

**DOI:** 10.1155/cjid/2148587

**Published:** 2026-03-04

**Authors:** Jingjing Xu, Chiwen Cheng, Kang Yu, Qing Wang, Yangyang Peng, Yanqing Li, Wen Yao, Yijie Pi, Shuhuan Yu, Zerui Han, Jing Wei, Tingtao Chen, Yifeng Yu

**Affiliations:** ^1^ Ophthalmic Center, The Second Affiliated Hospital, Jiangxi Medical College, Nanchang University, Nanchang, Jiangxi, China, ncu.edu.cn; ^2^ School of General Practice, Gannan Medical University, Ganzhou, Jiangxi, China, gmu.cn; ^3^ Jiangxi Medical College, Nanchang University, Nanchang, Jiangxi, China, ncu.edu.cn; ^4^ National Engineering Research Center for Bioengineering Drugs and the Technologies, Institute of Translational Medicine, Jiangxi Medical College, Nanchang University, Nanchang, Jiangxi, China, ncu.edu.cn

**Keywords:** dry eye, FS-LASIK, ocular surface microbiome, 16S rRNA

## Abstract

**Purpose:**

Given the limited evidence on ocular surface microbiota (OSM) changes after femtosecond laser‐assisted in situ keratomileusis (FS‐LASIK) and their link to dry eye (DE), this study aimed to compare microbial profiles in patients with and without postoperative DE, offering a basis for early detection and targeted treatment.

**Methods:**

Patients undergoing FS‐LASIK were evaluated 3 months postoperatively and stratified into DE (*N* = 30) and non‐DE groups (*N* = 30) based on ocular surface disease index (OSDI), TBUT, and the Schirmer I test. Corneal nerve alterations were assessed using IVCM. Conjunctival sac samples were collected pre‐ and postoperatively, and microbiota profiles were analyzed via 16S rRNA sequencing.

**Results:**

Comparative analysis of the OSM revealed significantly higher α‐diversity in the DE group compared to the NDE group. At the phylum level, a decrease in Proteobacteria and an increase in Bacteroidetes were observed. At the genus level, *Veillonella*, *Streptococcus*, and *Aggregatibacter* were enriched, whereas *Pseudomonas* and *Lactobacillus* were depleted. The abundance of *Staphylococcus* was positively correlated with OSDI scores and negatively correlated with corneal nerve fiber length (CNFL), corneal nerve branch density (CNBD), corneal nerve fiber area (CNFA), and corneal nerve fractal dimension (CFracDim) (all *p* < 0.05). In contrast, *Lactobacillus* was positively associated with the Schirmer I test values and corneal nerve fiber density (CNFD) (*p* < 0.05). At baseline, patients in the DE group exhibited lower abundances of Cyanobacteria and *Acinetobacter* but higher levels of Verrucomicrobia and *Akkermansia*. Postoperative within‐group comparisons further showed that, relative to baseline, the DE group had increased abundances of *Staphylococcus*, *Veillonella*, *Ralstonia*, and *Aggregatibacter*, along with decreased levels of *Pseudomonas*, *Corynebacterium*, and *Helicobacter*.

**Conclusions:**

In summary, dysbiosis between the *Staphylococcus* and *Lactobacillus* genera may represent both a biomarker of DE susceptibility and a therapeutic target. Additionally, the preoperative microbial composition may influence postoperative dynamics and DE risk.

**Trial Registration:**

ClinicalTrials.gov identifier: NCT06448468.

## 1. Introduction

With myopia prevalence surpassing 80% among East Asian adolescents, demand for refractive correction is soaring [1]. In China, femtosecond laser‐assisted in situ keratomileusis (FS‐LASIK) is the gold‐standard procedure but induces postoperative dry eye (DE) in 20%–55% of cases [2], marked by tear instability, neuropathic pain, and corneal hypoesthesia [3, 4]. Chronic DES triggers epithelial–stromal remodeling via mechano‐inflammatory pathways, leading to refractive regression and lasting visual decline [2, 5]. Understanding DE pathogenesis post‐FS‐LASIK is thus essential for targeted therapies to restore ocular surface integrity.

Postoperative DE is primarily attributed to laser‐induced corneal nerve damage, impairing tear regulation [6]. However, high‐throughput sequencing challenges the “sterile ocular surface” view, linking ocular surface microbiota (OSM) dysbiosis to DE pathogenesis [7, 8]. Studies report elevated opportunistic pathogens (e.g., *Staphylococcus epidermidis*) in DE patients compared to healthy controls [9–11]. However, emerging evidence also suggests that a reduction in *S. epidermidis* may compromise ocular surface immunity and contribute to inflammation or infection susceptibility [12]. These seemingly contradictory findings highlight the dual role of *S. epidermidis* as both a commensal and a potential opportunistic pathogen, depending on host context, strain characteristics, and microbial community interactions. Ozkan et al. observed increased β‐diversity and a shift from commensal *Corynebacterium* to pathogenic *Staphylococcus* in meibomian gland dysfunction–related DE [13]. Zou et al. identified stage‐specific microbiome alterations, with *Pseudomonas* and *Bacillus* enriched in moderate and severe DE, respectively, both associated with heightened infection risk [14]. However, such microbial shifts may also be influenced by external factors, such as hypoxic conditions associated with contact lens wear, rather than DE severity alone. Yet, the impact of refractive surgery on OSM remains unclear. Emerging evidence links dysbiosis, particularly *Staphylococcus* overgrowth, to subclinical inflammation in DE [15]. Surgical trauma and perioperative medications may disrupt microbial homeostasis, initiating neuroinflammatory cascades that impair corneal nerves and tear stability, highlighting the need to explore FS‐LASIK–induced microbial remodeling.

In this prospective longitudinal study, we apply 16S rRNA sequencing to assess OSM changes in 60 FS‐LASIK patients by comparing preoperative and 3‐month postoperative profiles. Through correlating microbiome alterations with symptoms of DE, our objective is to explore the microbiome‐associated mechanisms underlying postoperative DE, offering insights for precise management strategies of postoperative DE and enhanced refractive surgery outcomes.

## 2. Methods

### 2.1. Patients

This study prospectively enrolled patients undergoing FS‐LASIK between April and September 2024. All participants completed standardized DE evaluations both preoperatively and 3 months postoperatively. Based on predefined criteria, subjects were categorized into two groups: (1) FS‐LASIK–induced DE and (2) non‐dry eye (NDE). The study protocol was approved by the Ethics Committee of the Second Affiliated Hospital of Nanchang University ([2024] No. 34) and conducted in accordance with the principles of the Declaration of Helsinki.

Inclusion criteria were as follows: (1) age ≥ 18 years; no history of ocular/systemic disease, trauma, or prior surgery (except FS‐LASIK); (2) prophylactic antibiotic and anti‐inflammatory eye drops used from 3 days before to 1 month after surgery; no use of additional antibiotics, immunosuppressants, or preservative‐containing drops; (3) DE diagnosis required: (a) subjective symptoms: ocular surface disease index (OSDI) score ≥ 13 and (b) objective signs: TBUT > 5 s and ≤ 10 s and the Schirmer I test > 5 mm and ≤ 10 mm/5 min, with corneal fluorescein staining score ≥ 5 [16, 17].

The exclusion criteria were as follows: (1) history of severe ocular conditions (e.g., corneal disease, glaucoma, retinal detachment, autoimmune disease, trauma, or surgery) and (2) severe preoperative DE: TBUT < 2 s, extensive epithelial defects (≥ 2 quadrants), or fluorescein staining ≥ 30.

### 2.2. Confocal Microscopy and Image Analysis

In vivo confocal microscopy (IVCM; Heidelberg Retina Tomograph III with Rostock Cornea Module, Heidelberg Engineering GmbH, Germany) was performed using standardized protocols to assess corneal nerves [18, 19]. Scans focused on the corneal apex, with proper eye positioning maintained by an experienced operator (YY). Three high‐quality images—selected by two masked observers (JX and CC) based on clarity, contrast, and absence of artifacts—were analyzed using ACCMetrics software (University of Manchester, UK). The following parameters were automatically quantified: (1) corneal nerve fiber density (CNFD, no./mm^2^), (2) nerve fiber length (CNFL, mm/mm^2^), (3) branch density (CNBD, no./mm^2^), (4) total branch density (CTBD, no./mm^2^), (5) fiber area (CNFA, mm^2^/mm^2^), (6) fiber width (CNFW, mm/mm^2^), and (7) fractal dimension (CFracDim, reflecting spatial nerve loss). Mean values were calculated from the three images for each parameter.

### 2.3. Sample Collection

Ocular surface swabs were collected in a sterile prep room by trained ophthalmologists. After hand disinfection and gloving, topical anesthesia (proparacaine hydrochloride) was applied. After 1–3 min, with the patient gazing upward, a sterile polyester swab was gently rotated twice along the inferior conjunctival fornix. The swab was immediately sealed in a DNA/RNA‐free cryotube, transported on dry ice, and stored at −80°C until centralized nucleic acid extraction.

Samples were obtained at two defined time points to minimize potential confounding factors: (1) Preoperative specimens were collected before the administration of any prophylactic topical antibiotics, thereby preserving the native microbial community and (2) Postoperative specimens were collected 3 months following FS‐LASIK, at which point all perioperative topical medications, including corticosteroids and antibiotics, had been discontinued for a minimum of 8 weeks, ensuring a washout period sufficient to eliminate pharmacological effects on the OSM.

### 2.4. DNA Extraction and 16S rRNA Sequencing

Total genomic DNA was extracted using the MagBeads FastDNA Kit for Soil (116564384; MP Biomedicals, CA, USA) according to the manufacturer’s instructions and stored at −20°C until further analysis. DNA quantity and quality were assessed using a NanoDrop NC2000 spectrophotometer (Thermo Fisher Scientific, Waltham, MA, USA) and agarose gel electrophoresis, respectively. The V4 region of the bacterial 16S rRNA gene was amplified by PCR using the forward primer 520F (5′‐AYTGGGYDTAAAGNG‐3′) and the reverse primer 802R (5′‐TACNVGGGTATCTAATCC‐3′). Sample‐specific 7‐bp barcodes were incorporated into the primers for multiplex sequencing. The PCR reaction mixture included 5 μL of 5× buffer, 0.25 μL of FastPfu DNA polymerase (5 U/μL), 2 μL of dNTPs (2.5 mM), 1 μL each of forward and reverse primers (10 μM), 1 μL of DNA template, and 14.75 μL of ddH_2_O, for a total volume of 25 μL. Thermal cycling conditions were as follows: initial denaturation at 98°C for 5 min; 25 cycles of denaturation at 98°C for 30 s, annealing at 53°C for 30 s, and extension at 72°C for 45 s; followed by a final extension at 72°C for 5 min. PCR amplicons were purified using Vazyme VAHTSTM DNA Clean Beads (Vazyme, Nanjing, China) and quantified with the Quant‐iT PicoGreen dsDNA Assay Kit (Invitrogen, Carlsbad, CA, USA). After individual quantification, amplicons were pooled in equal amounts. Paired‐end 2250 bp sequencing was conducted on the Illumina NovaSeq platform using the NovaSeq 6000 SP Reagent Kit (500 cycles) at Shanghai Personal Biotechnology Co., Ltd. (Shanghai, China).

### 2.5. Statistical Analysis

Statistical analyses were conducted using SPSS 26.0 (IBM Corp.) and *R* 4.2.1. Normality of continuous variables was assessed with the Shapiro–Wilk test. Data are presented as mean ± SD for normally distributed variables and median (interquartile range) for non‐normal distributions. Between‐group comparisons used independent *t*‐tests or Mann–Whitney *U* tests, as appropriate. Paired intra‐group differences were analyzed with the Wilcoxon signed‐rank test. Categorical variables were reported as counts (%) and compared using the chi‐square or Fisher’s exact test. Correlations were assessed using Spearman’s method. A two‐tailed *p* value < 0.05 was considered statistically significant.

## 3. Results

### 3.1. Patients’ Baseline Characteristics

**FIGURE 1:**
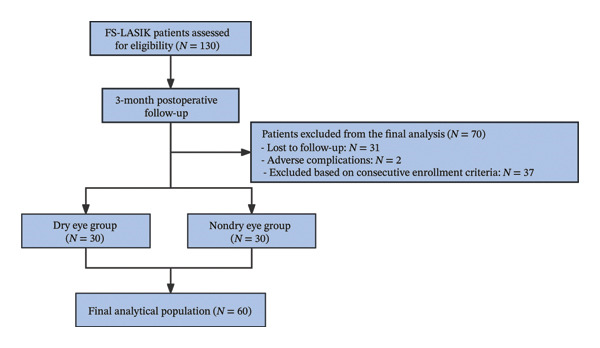
Patient recruitment and grouping flowchart.

A total of 130 FS‐LASIK patients were initially enrolled. At the 3‐month follow‐up, 33 were excluded—31 lost to follow‐up and 2 with surgical complications. To ensure balanced comparisons and reduce variability, only the first 30 eligible patients per group were included; 37 additional qualified patients were excluded due to sample size limits (Figure 1). Based on predefined criteria, participants were categorized into two groups: (1) the DE group and (2) the NDE group.

The DE and NDE groups were comparable in baseline characteristics (Table 1), with similar median ages (24 vs. 26 years) and gender distribution (46.7% vs. 56.7% female). There were no significant differences in preoperative parameters, including spherical equivalent, TBUT, the Schirmer I test, OSDI scores, or corneal nerve morphology. The consistency of these baseline data confirms robust group matching for subsequent postoperative analyses.

**TABLE 1:** Baseline patient demographics and characteristics.

Characteristics	Pre_DE (*N* = 30)	Pre_NDE (*N* = 30)	t/Z/*χ*2	*p* value
Age, y	24 (21.75,27.25)	26 (22.75,30.00)	−1.009	0.313
Gender, *n* (%)	—	—	0.601	0.438
Male	16 (53.3)	13 (43.3)	—	—
Female	14 (46.7)	17 (56.7)	—	—
SE, D	−6.01 ± 1.25	−6.03 ± 1.29	0.051	0.960
TBUT, s	7.70 (7.05,9.02)	7.86 (7.44, 9.26)	−1.383	0.167
Schirmer I test, mm/5 min	14.50 (11.00,16.00)	12.50 (10.00, 15.00)	−1.526	0.127
OSDI	4.17 (0.00,6.25)	4.17 (2.08, 4.17)	−0.137	0.891
CNFD, no./mm^2^	31.25 (25.00,33.33)	27.08 (22.92, 33.33)	−0.944	0.345
CNBD, no./mm^2^	36.02 (21.75,52.36)	30.48 (20.98, 43.09)	−0.843	0.399
CNFL, mm/mm^2^	16.74 ± 3.40	16.66 ± 3.57	0.084	0.934
CTBD, no./mm^2^	67.70 (44.23,101.04)	60.41 (49.48, 84.89)	−0.118	0.906
CNFA, mm^2^/mm^2^	0.007 ± 0.002	0.008 ± 0.002	−0.076	0.940
CNFW, mm/mm^2^	0.021 ± 0.001	0.021 ± 0.001	−0.461	0.646
CFracDim	1.51 (1.48,1.52)	1.50 (1.47, 1.52)	−0.399	0.690

*Note:* Data are presented as *n* (%), mean ± standard deviation, or median (interquartile range). Pre_DE, preoperative samples from patients who developed postoperative dry eye; Pre_NDE, preoperative samples from patients who did not develop postoperative dry eye. Statistical comparisons were performed using the Mann–Whitney *U* test for non‐normally distributed continuous variables, the independent samples *t*‐test for normally distributed continuous variables, and the chi‐square test for categorical variables.

Abbreviations: CFracDim = corneal fractal dimension, CNBD = corneal nerve branch density, CNFA = corneal nerve fiber area, CNFD = corneal nerve fiber density, CNFL = corneal nerve fiber length, CNFW = corneal nerve fiber width, CTBD = corneal total branch density, OSDI = ocular surface disease index, SE = spherical equivalent, TBUT = tear break‐up time.

### 3.2. Postoperative Ocular Surface and Nerve Changes After FS‐LASIK

At the 3‐month postoperative follow‐up, patients were categorized into DE and NDE groups based on subjective symptoms and objective signs (Table 2). Significant differences were observed between groups in TBUT, the Schirmer I test, and OSDI scores (all *p* < 0.001).

**TABLE 2:** The parameter variables of the postoperative DE group and NDE group.

Characteristics	DE (*N* = 30)	NDE (*N* = 30)	Z	*p* value
TBUT, s	4.83 (4.20,5.74)	7.02 (5.89,7.91)	−4.857	< 0.001^∗∗∗^
Schirmer I test, mm/5 min	4.00 (3.00,7.00)	12.0 (9.75,15.00)	−5.298	< 0.001^∗∗∗^
OSDI	18.75 (14.58,23.44)	6.25 (4.17,8.33)	−6.681	< 0.001^∗∗∗^

*Note:* Data are presented as median (interquartile range). Statistical comparisons were performed using the Mann–Whitney *U* test for continuous variables with non‐normal distribution.

Abbreviations: DE = dry eye, NDE = non‐dry eye, OSDI = ocular surface disease index, TBUT = tear break‐up time.

Significance levels: ^∗∗∗^ *p* < 0.001.

Postoperatively, the DE group exhibited significantly lower corneal nerve parameters compared to the NDE group, including CNFD, CNBD, CNFL, CTBD, and CFracDim (all *p* < 0.05; Figures 2(a), 2(b), 2(c), 2(d), 2(g)), whereas no significant differences were observed in CNFW (*p* = 0.617, Figure 2(e)) or CNFA (*p* = 0.101, Figure 2(f)). These reductions in nerve fiber density and complexity suggest more pronounced corneal neural impairment associated with postoperative DE.

FIGURE 2Changes in corneal nerve parameters between the DE and NDE groups. (a) CNFD, corneal nerve fiber density; (b) CNBD, corneal nerve branch density; (c) CNFL, corneal nerve fiber length; (d) CTBD, corneal total branch density; (e) CNFW, corneal nerve fiber width; (f) CNFA, corneal nerve fiber area; (g) CFracDim, corneal fractal dimension. DE: dry eye group (*N* = 30); NDE: non‐dry eye group (*N* = 30). Statistical comparisons between groups were performed using the Mann–Whitney *U* test. Significance levels: ^∗^ *p* < 0.05, ^∗∗^ *p* < 0.01, ^∗∗∗^ *p* < 0.001, ^∗∗∗∗^ *p* < 0.0001; ns: nonsignificant.

**Figure:**
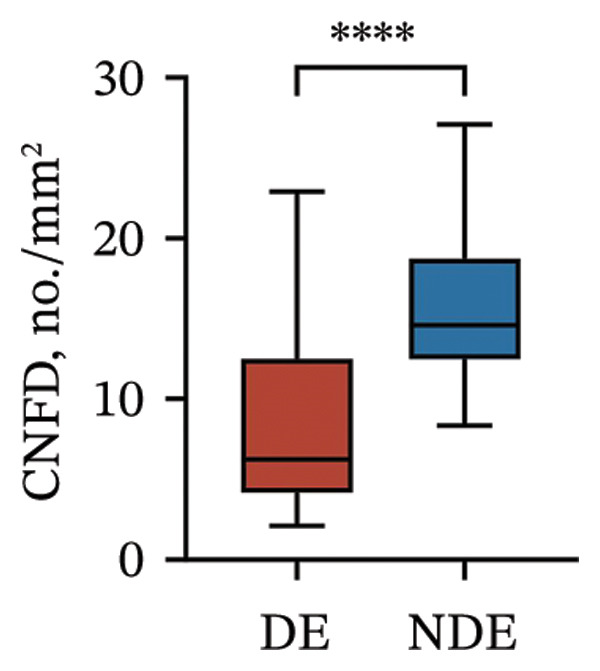
(a)

**Figure:**
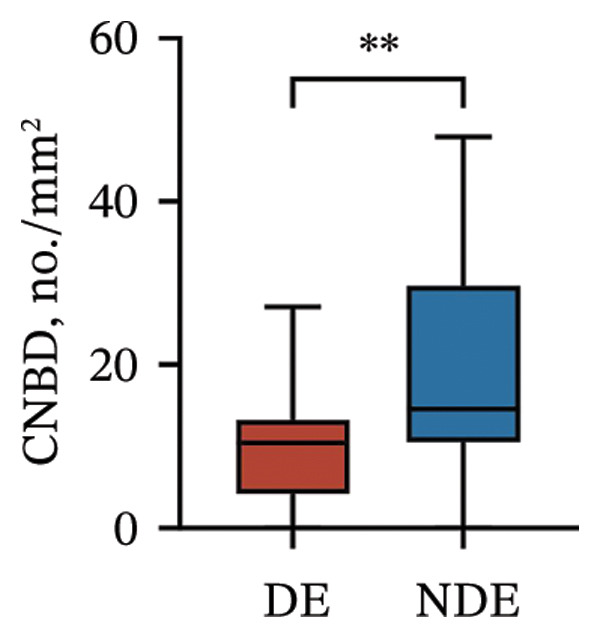
(b)

**Figure:**
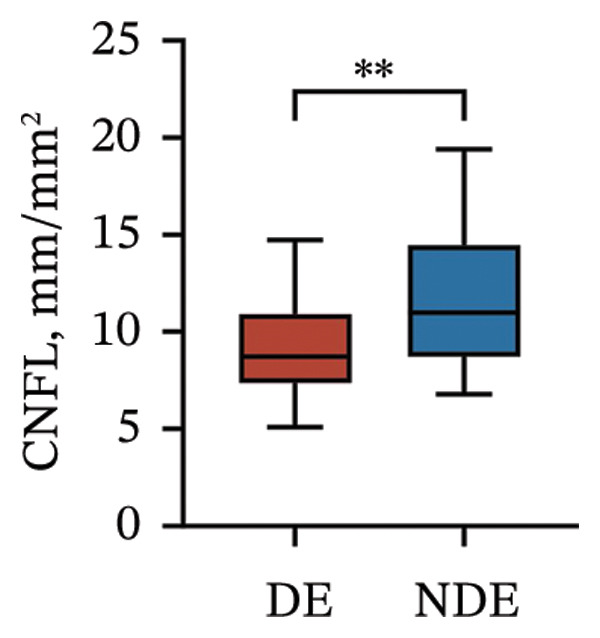
(c)

**Figure:**
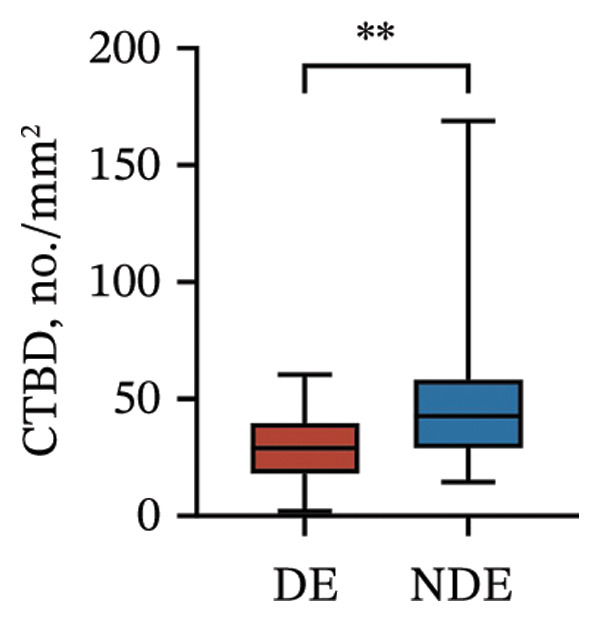
(d)

**Figure:**
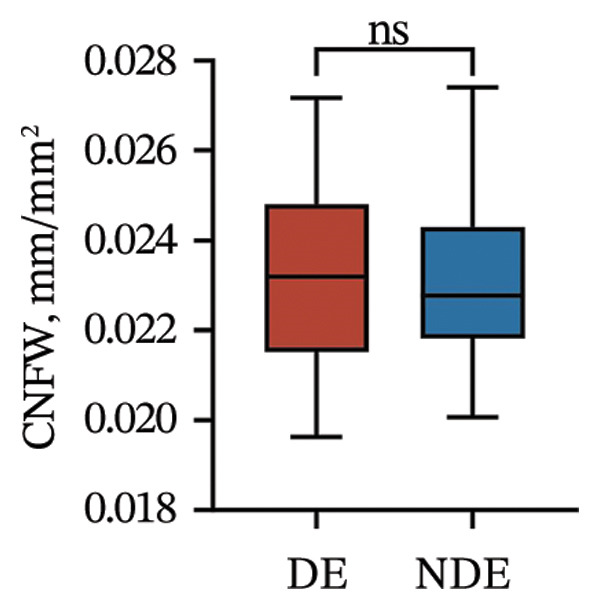
(e)

**Figure:**
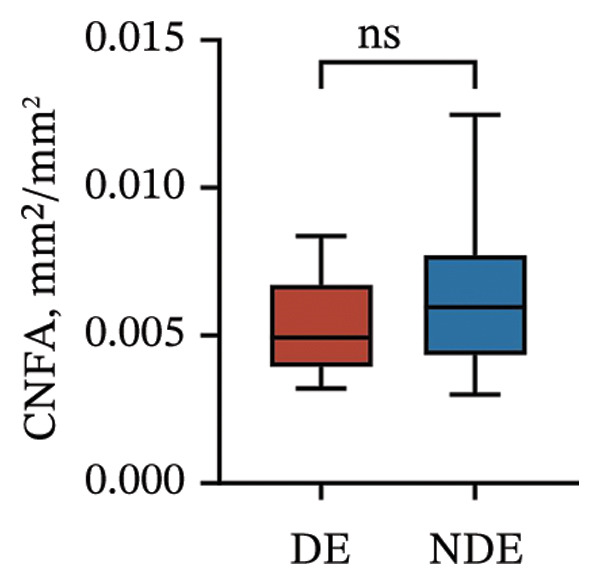
(f)

**Figure:**
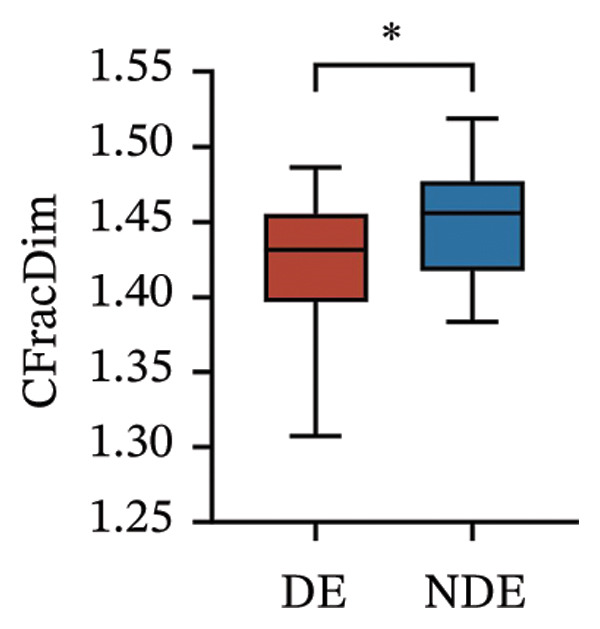
(g)

### 3.3. Distinct Microbiome Profiles in FS‐LASIK–Induced DE

To investigate the microbial basis of DE development after FS‐LASIK, we performed 16S rRNA sequencing on ocular surface samples from postoperative DE patients and NDE controls. Rarefaction curves indicated sufficient sequencing depth, with gene richness reaching saturation in both groups (Figure 3(a)). Alpha diversity was significantly higher in the DE group, as reflected by an elevated Chao1 index (*p* = 0.02; Figure 3(b)), suggesting increased microbial richness. Principal coordinate analysis (PCoA) based on Jaccard distance revealed significant beta diversity differences, indicating distinct microbial community structures (Figure 3(d)).

FIGURE 3Distinct ocular surface microbiota profiles in DE versus NDE patients following FS‐LASIK. (a) Rarefaction curves showing sequencing depth and microbial richness. (b) Alpha diversity is assessed by the Chao1 index. (c) Venn diagram illustrating shared and unique operational taxonomic units (OTUs) between DE and NDE groups. (d) Principal coordinates analysis (PCoA) of β‐diversity based on Jaccard distances, highlighting distinct microbial community structures. (e) Linear discriminant analysis effect size (LEfSe) identifying differentially abundant taxa. (f) Stacked bar chart of phylum‐level taxonomic distributions. (g, h) Relative abundances of Proteobacteria and Bacteroidetes in DE versus NDE groups. (i) Genus‐level taxonomic distribution (top 20 genera). (j–n) Relative abundances of selected genera: *Pseudomonas*, *Veillonella*, *Streptococcus*, *Aggregatibacter*, and *Lactobacillus*. (o) Heatmap showing differences in ocular surface microbiota profiles between DE and NDE groups. Statistical significance was assessed using the Mann–Whitney *U* test. Significance levels:^∗^ *p* < 0.05, ^∗∗^ *p* < 0.01.

**Figure:**
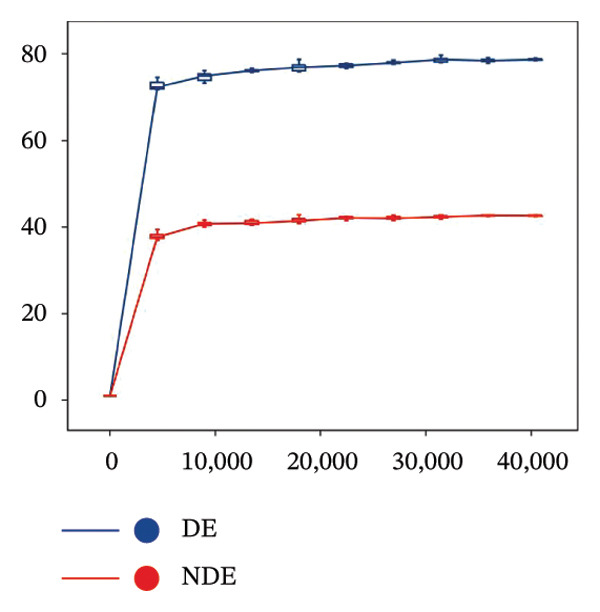
(a)

**Figure:**
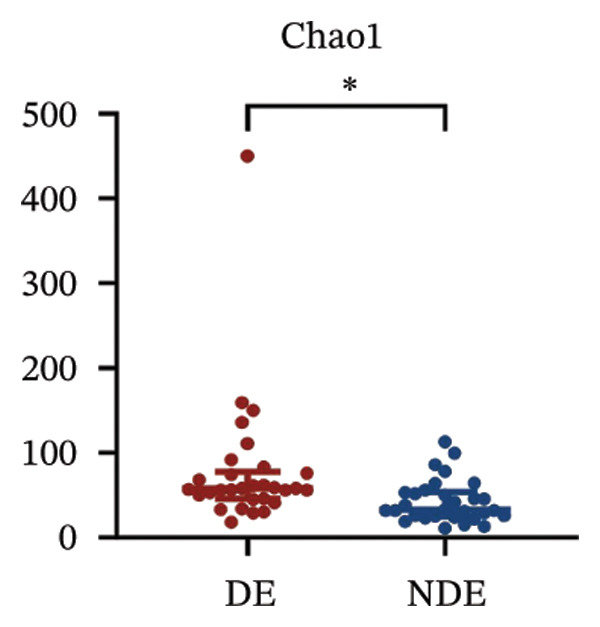
(b)

**Figure:**
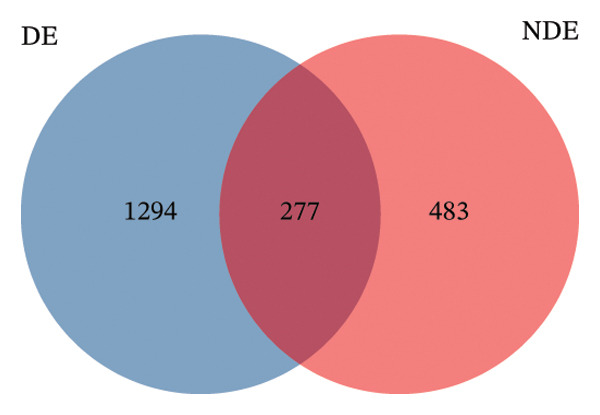
(c)

**Figure:**
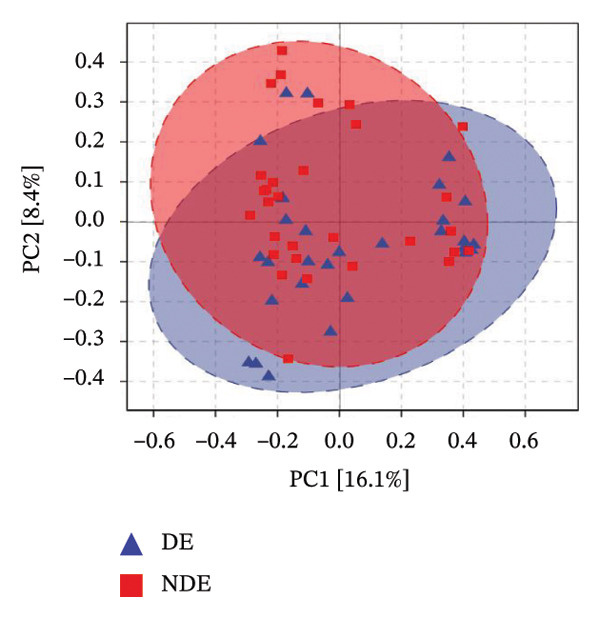
(d)

**Figure:**
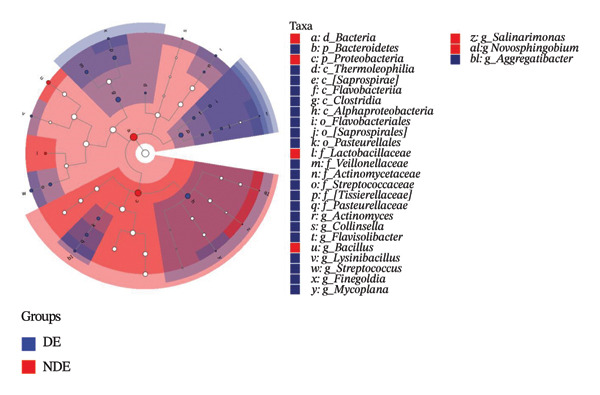
(e)

**Figure:**
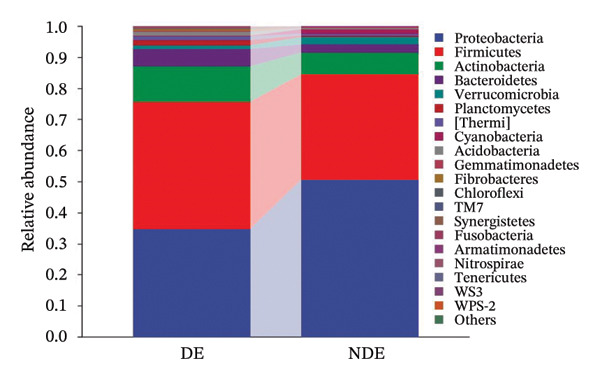
(f)

**Figure:**
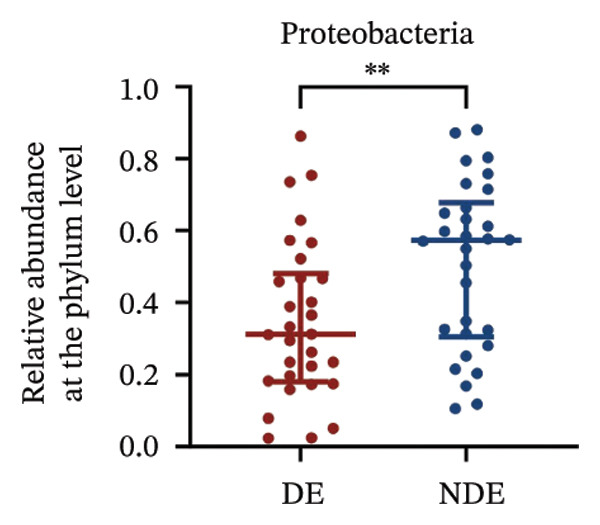
(g)

**Figure:**
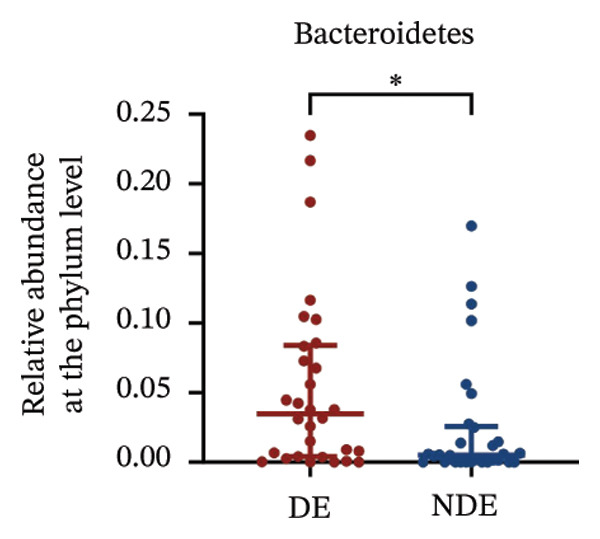
(h)

**Figure:**
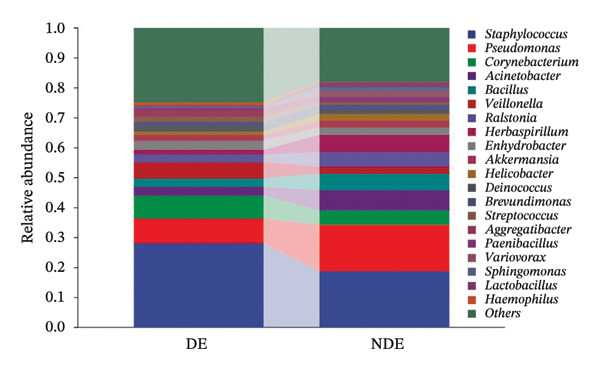
(i)

**Figure:**
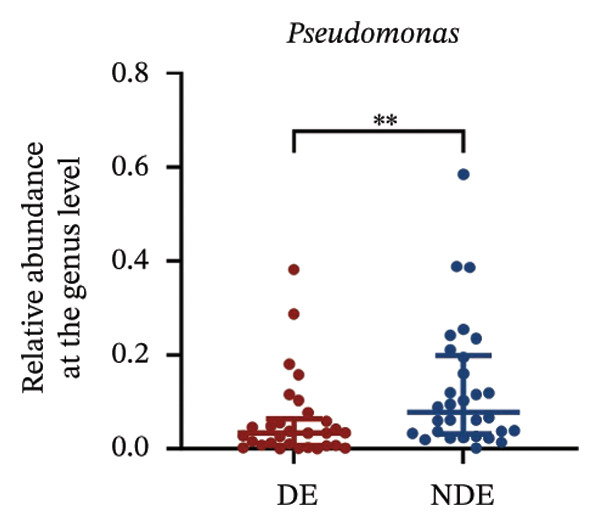
(j)

**Figure:**
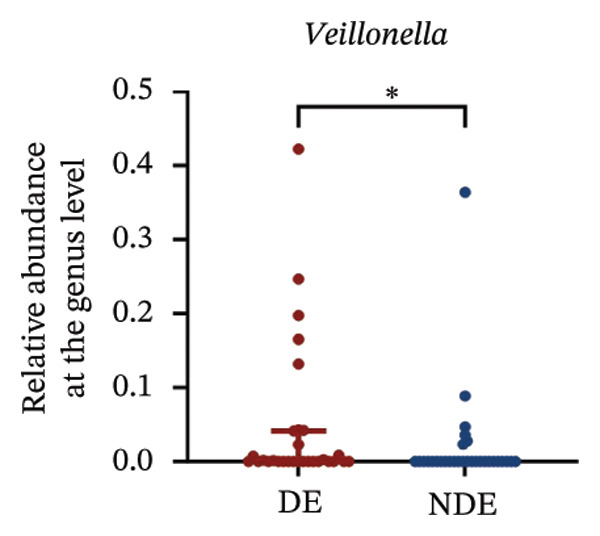
(k)

**Figure:**
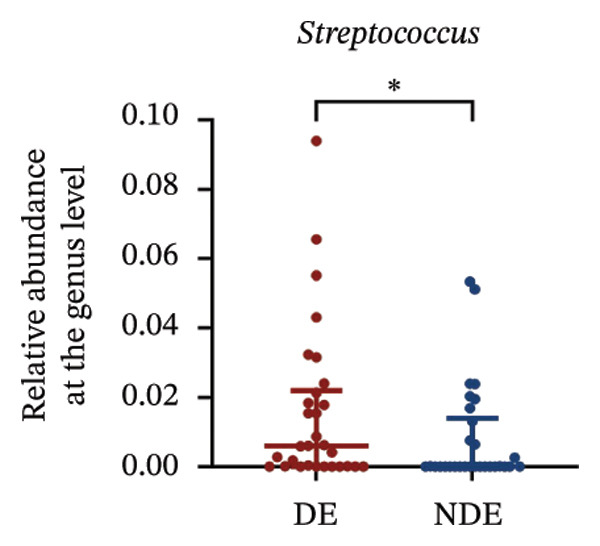
(l)

**Figure:**
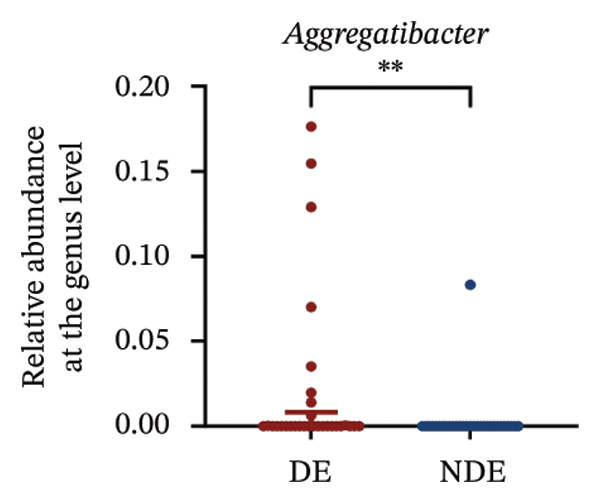
(m)

**Figure:**
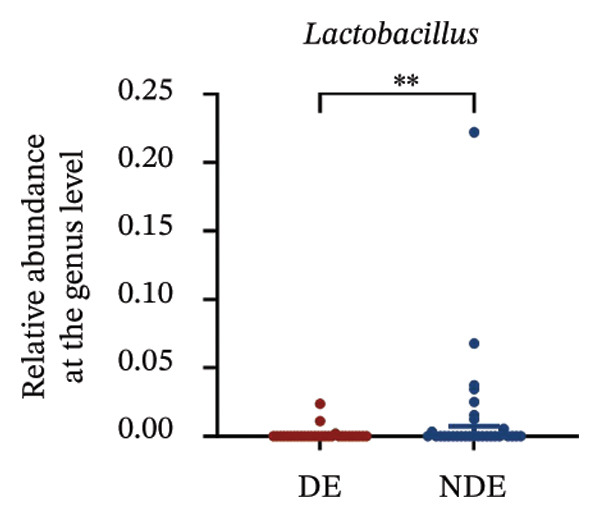
(n)

**Figure:**
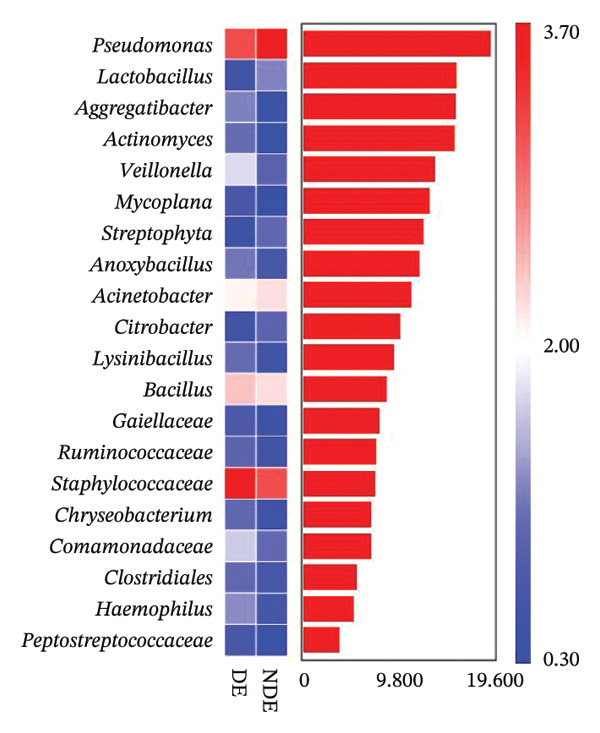
(o)

After quality control, 2054 operational taxonomic units (OTUs) were identified, with 277 shared between groups (Figure 3(c)). The DE group exhibited a higher total OTU count and microbial load. LEfSe analysis confirmed significant compositional differences between groups (Figure 3(e)). These findings highlight significant alterations in both α‐ and β‐diversity in DE patients, supporting a role for microbiota dysbiosis in postoperative DE.

At the phylum level, Proteobacteria, Firmicutes, Actinobacteria, and Bacteroidetes predominated (Figure 3(f)). DE patients showed reduced Proteobacteria and increased Bacteroidetes abundance compared to NDE controls (Figures 3(g), 3(h)). Genus‐level analysis identified *Staphylococcus*, *Pseudomonas*, *Corynebacterium*, and *Acinetobacter* as dominant genera in the overall cohort (Figure 3(i)). In the DE group, relative abundances of *Veillonella*, *Streptococcus*, and *Aggregatibacter* were significantly elevated (Figures 3(k), 3(l), 3(m)), while levels of *Pseudomonas* and *Lactobacillus* were significantly reduced (Figures 3(j), 3(n)). Heatmap analysis revealed clear group‐level differences in microbial composition (Figure 3(o)).

### 3.4. Genus‐Level Associations With DE Severity and Corneal Nerve Changes

**FIGURE 4:**
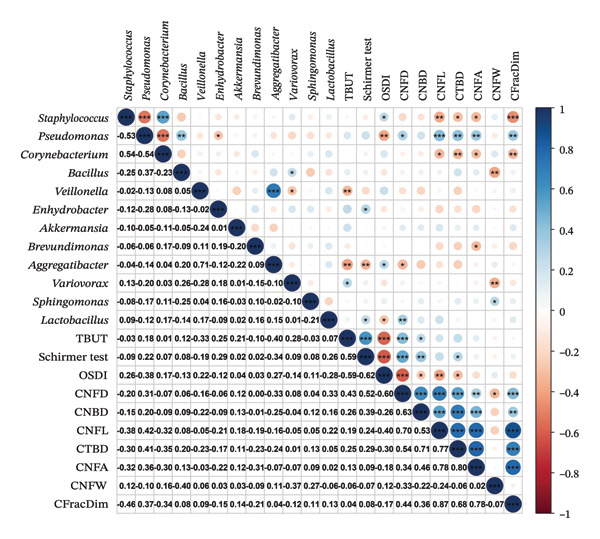
Correlation analysis between ocular surface microbiota and dry eye/corneal nerve parameters. Spearman correlation analysis revealed associations between genus‐level microbial abundance and key postoperative clinical indicators, including dry eye symptoms (TBUT, OSDI, Schirmer I test) and corneal nerve parameters (CNFD, CNBD, CNFL, CTBD, CNFA, CNFW, CFracDim). Blue indicates positive correlations, and red indicates negative correlations. Significance levels: ^∗^ *p* < 0.05, ^∗∗^ *p* < 0.01, ^∗∗∗^ *p* < 0.001.

Spearman correlation analysis was performed to assess associations between postoperative OSM (genus level) and key clinical parameters, including DE symptoms (OSDI score, Schirmer I test) and corneal nerve metrics (CNFL, CTBD, CNFA, CFracDim) (Figure 4). Clinically, *Staphylococcus* abundance was positively correlated with OSDI scores (*ρ* = 0.26, *p* < 0.05), but negatively correlated with all assessed corneal nerve parameters (CNFL: *ρ* = −0.38; CTBD: *ρ* = −0.30; CNFA: *ρ* = −0.32; CFracDim: *ρ* = −0.46; all *p* < 0.05). *Corynebacterium* showed similar trends, whereas *Pseudomonas* displayed inverse associations. Notably, *Lactobacillus* abundance was inversely correlated with OSDI severity (*ρ* = −0.28, *p* < 0.05), and positively associated with Schirmer I values (*ρ* = 0.26) and CNFD (*ρ* = 0.33; both *p* < 0.05).

### 3.5. Baseline OSM Differences Between DE and NDE Patients Before FS‐LASIK

A comprehensive baseline comparison of OSM between the DE and NDE groups was conducted before FS‐LASIK (Figure 5). Alpha diversity (Chao1 index) showed no significant difference in species richness (*p* > 0.05; Figure 5(b)), and PCoA based on Bray–Curtis distances revealed overlapping beta diversity, indicating similar microbial structures (Figure 5(d)). A total of 1436 OTUs were detected, with 228 shared between groups (Figure 5(c)). Although the DE group showed a nonsignificant trend toward higher OTU richness and bacterial load, LEfSe analysis indicated only modest compositional differences (Figure 5(e)).

FIGURE 5Preoperative ocular surface microbiota profiles in patients with and without postoperative dry eye after FS‐LASIK. (a) Rarefaction curves showing microbial richness. (b) Alpha diversity (Chao1 index). (c) Venn diagram of shared and unique OTUs. (d) PCoA of β‐diversity based on Jaccard distance. (e) LEfSe analysis identifying differentially abundant taxa. (f) Phylum‐level taxonomic composition (stacked bar chart). (g–h) relative abundances of Cyanobacteria and Verrucomicrobia. (i) Genus‐level composition (top 20 genera). (j–k) relative abundances of *Akkermansia* and *Acinetobacter*. (l) Genus‐level heatmap showing intergroup differences. Pre_DE, preoperative samples from patients who developed postoperative dry eye (*N* = 30); Pre_NDE, preoperative samples from patients who did not develop postoperative dry eye (*N* = 30). Statistical significance was assessed using the Mann–Whitney *U* test. Significance levels: ^∗^ *p* < 0.05, ^∗∗^ *p* < 0.01, ns: nonsignificant.

**Figure:**
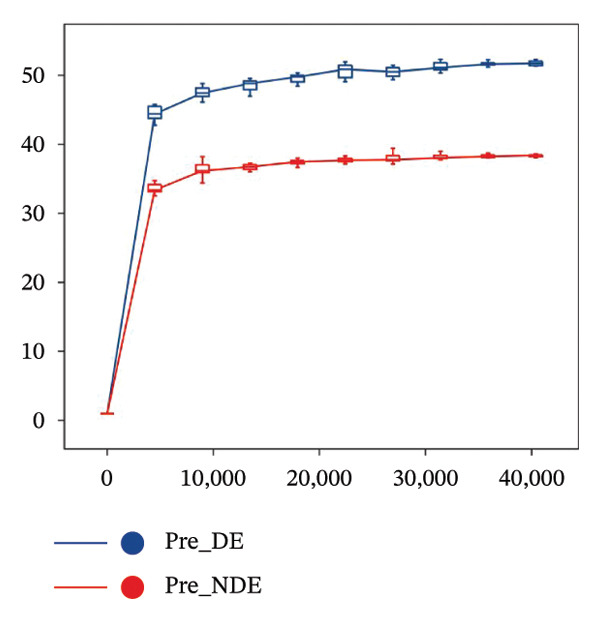
(a)

**Figure:**
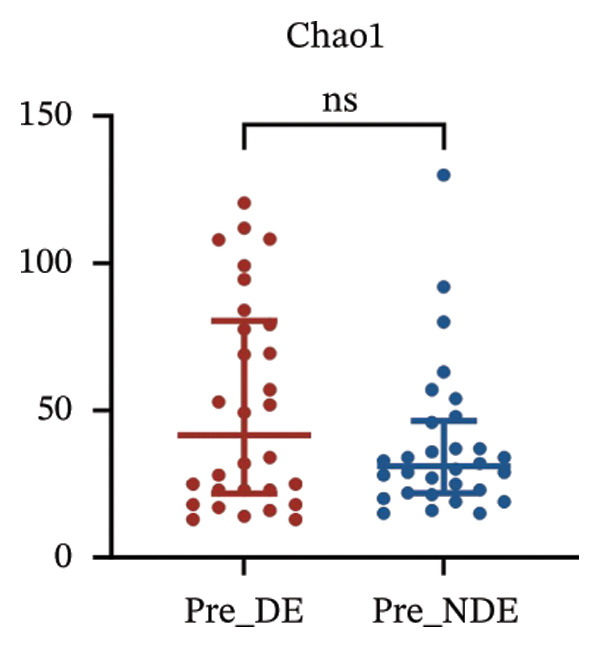
(b)

**Figure:**
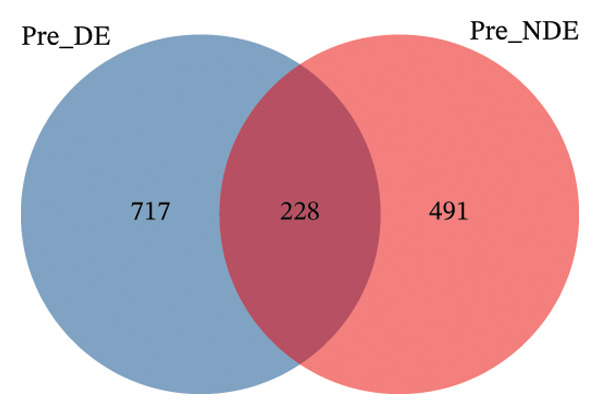
(c)

**Figure:**
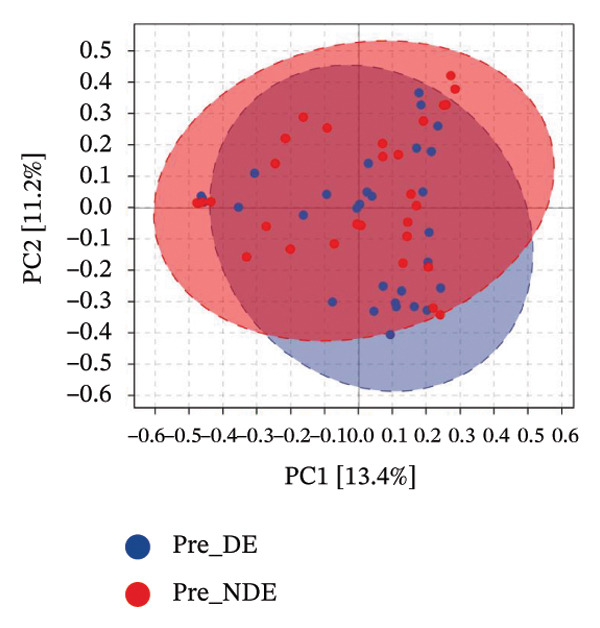
(d)

**Figure:**
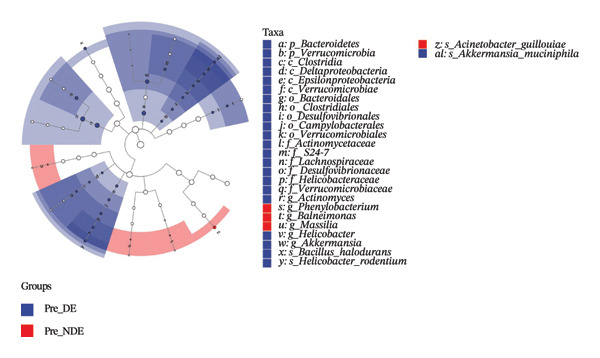
(e)

**Figure:**
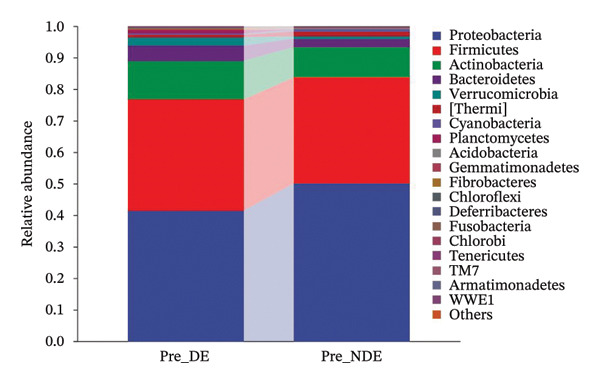
(f)

**Figure:**
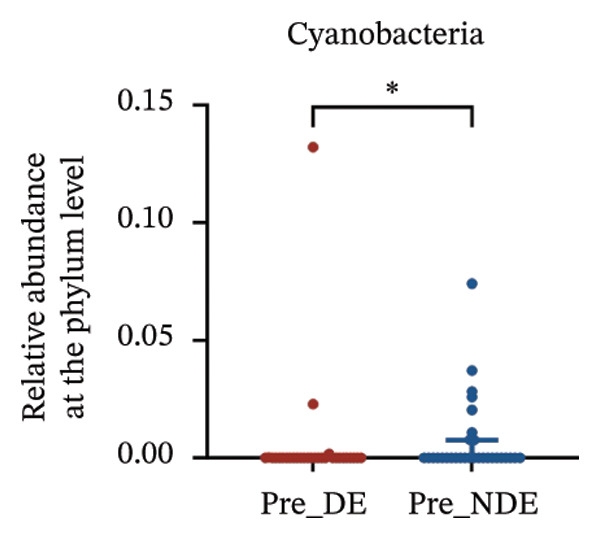
(g)

**Figure:**
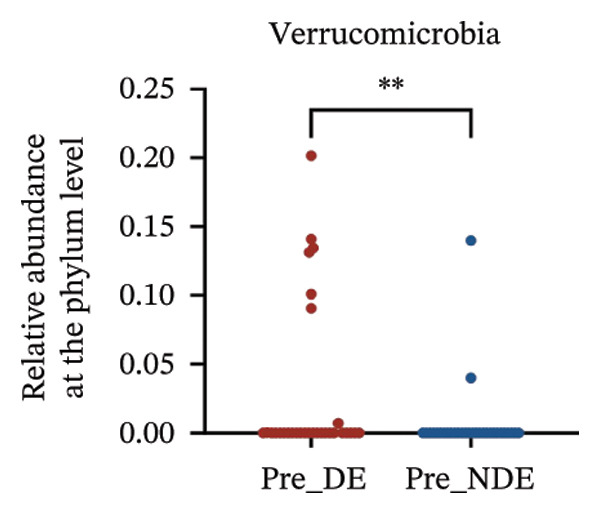
(h)

**Figure:**
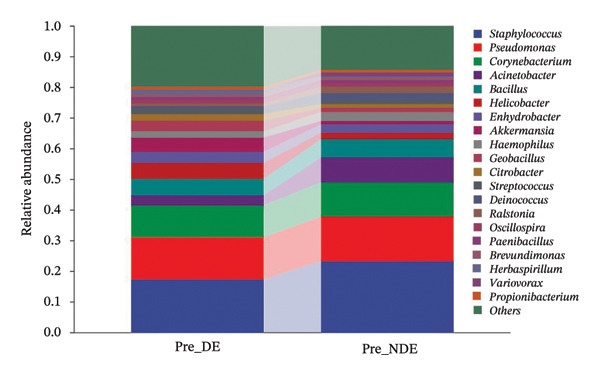
(i)

**Figure:**
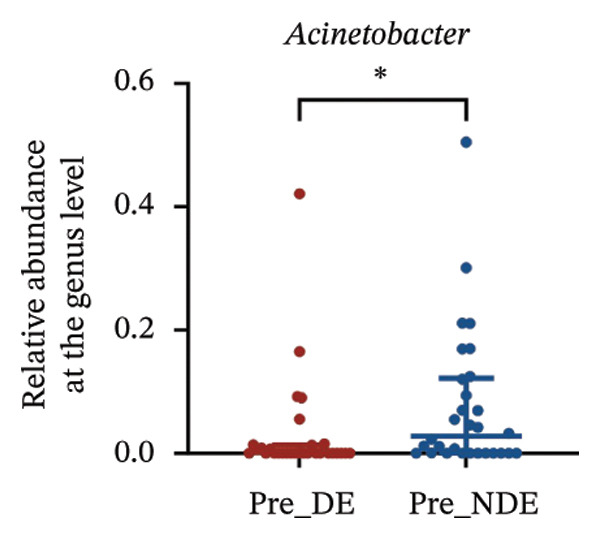
(j)

**Figure:**
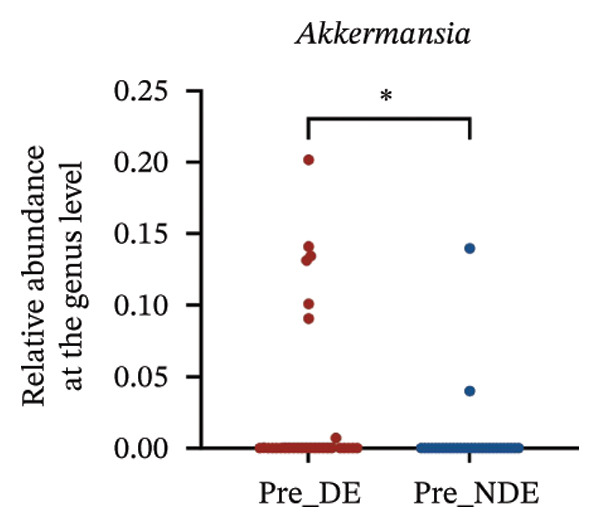
(k)

**Figure:**
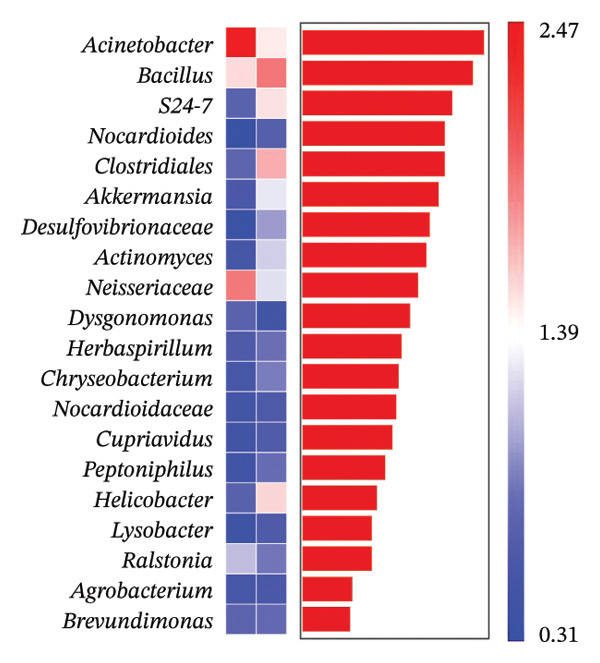
(l)

At the phylum level, Cyanobacteria were reduced (*p* < 0.05) and Verrucomicrobia increased (*p* < 0.01) in the DE group (Figures 5(g), 5(h)). Genus‐level analysis revealed decreased *Acinetobacter* and increased *Akkermansia* in DE patients (both *p* < 0.05; Figures 5(j), 5(k)). Heatmap visualization further supported subtle baseline differences between groups (Figure 5(l)).

### 3.6. Longitudinal Microbiota Changes in DE and NDE Groups After FS‐LASIK

To assess FS‐LASIK‐induced changes in OSM, we conducted longitudinal profiling in DE and NDE patients. While phylum‐level composition remained largely stable in both groups, significant genus‐level shifts were observed. In the DE cohort, postoperative increases in *Staphylococcus*, *Ralstonia*, *Veillonella*, and *Aggregatibacter* (*p* < 0.05) were accompanied by reductions in *Pseudomonas*, *Corynebacterium*, and *Helicobacter* (Figures 6(a), 6(b), 6(c), 6(d), 6(e), 6(f), 6(g), 6(h)), indicating ecological disruption potentially linked to immune imbalance. Notably, the relative abundance of *Staphylococcus* increased significantly in DE patients at 3 months postoperatively compared to baseline (*p* < 0.05). Although *Staphylococcus* also trended upwards in the NDE group, this change was not statistically significant, suggesting that *Staphylococcus* expansion is more strongly associated with FS‐LASIK‐induced DE.

FIGURE 6Within‐group comparison of pre‐ and postoperative ocular surface microbiota in FS‐LASIK dry eye and non‐dry eye patients. (a) Stacked bar plots showing genus‐level taxonomic composition in the DE group before and after FS‐LASIK. (b–h) Changes in the relative abundance of *Staphylococcus*, *Pseudomonas*, *Corynebacterium*, *Helicobacter*, *Veillonella*, *Ralstonia*, and *Aggregatibacter* before and after surgery. (i) Stacked bar plots showing genus‐level taxonomic composition in the NDE group before and after FS‐LASIK. (j–l) Changes in the relative abundance of *Corynebacterium*, *Herbaspirillum*, and *Ralstonia* before and after surgery. Post_DE: postoperative samples from the same patients diagnosed with dry eye after FS‐LASIK (*N* = 30). Post_NDE: Postoperative samples from the same patients without dry eye after FS‐LASIK (*N* = 30). Statistical comparisons were performed using the Wilcoxon signed‐rank test. Significance levels: ^∗^ *p* < 0.05, ^∗∗^ *p* < 0.01, ^∗∗∗^ *p* < 0.001.

**Figure:**
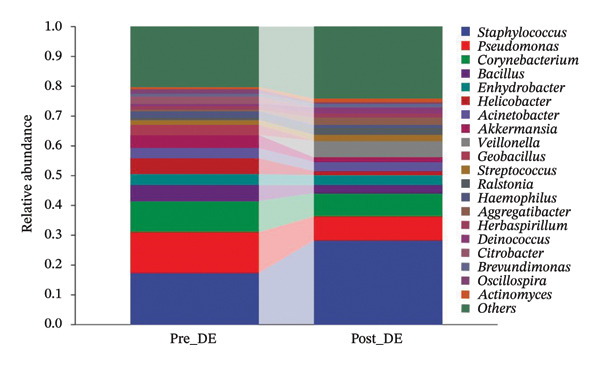
(a)

**Figure:**
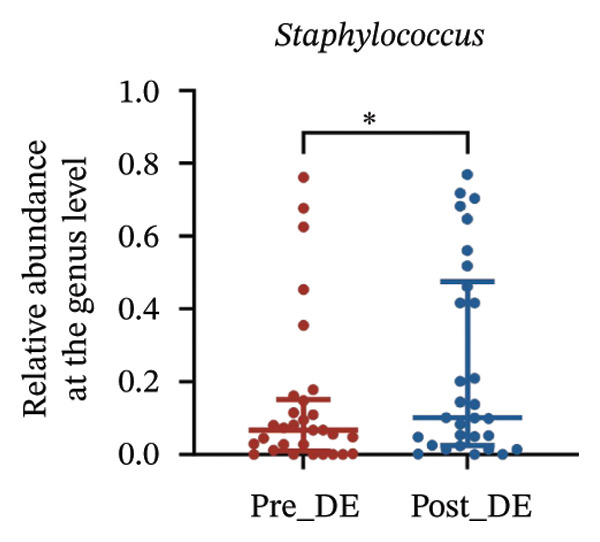
(b)

**Figure:**
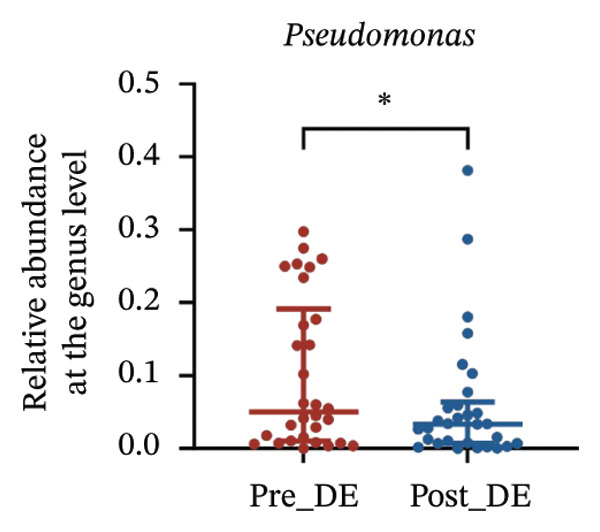
(c)

**Figure:**
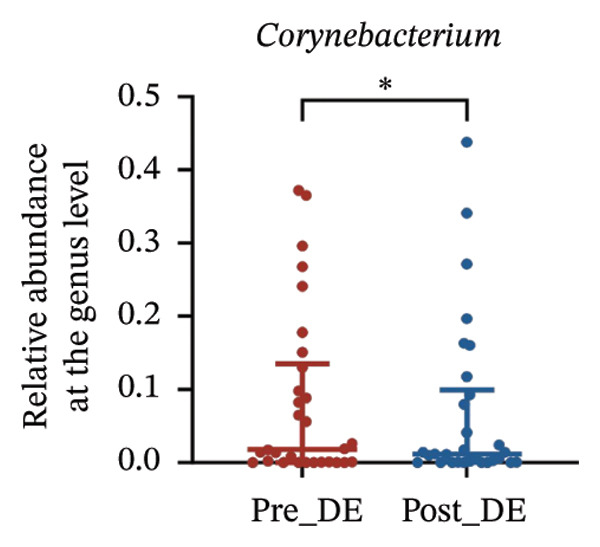
(d)

**Figure:**
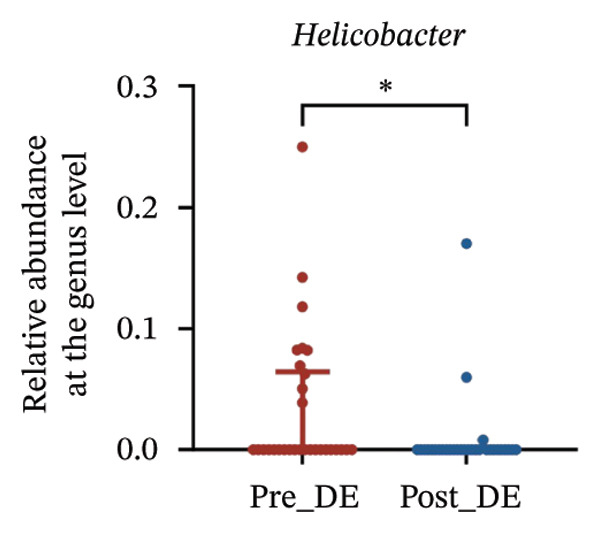
(e)

**Figure:**
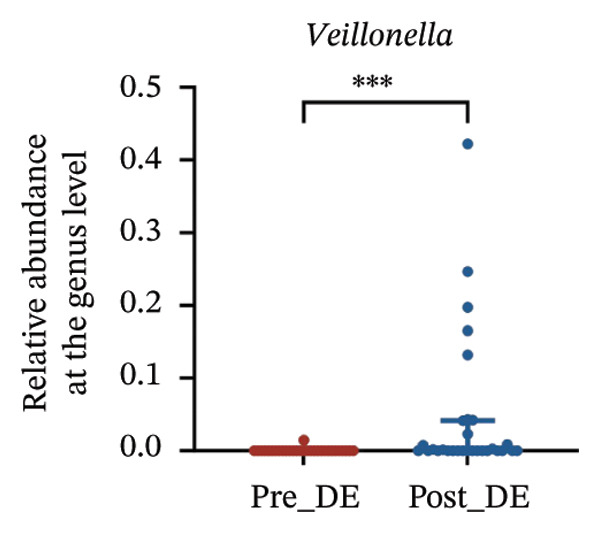
(f)

**Figure:**
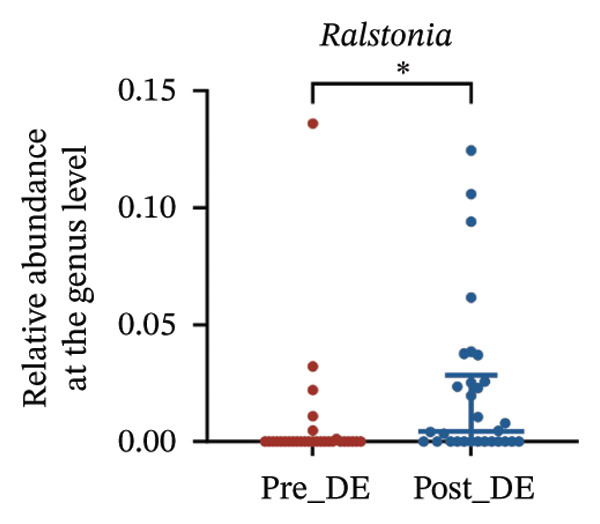
(g)

**Figure:**
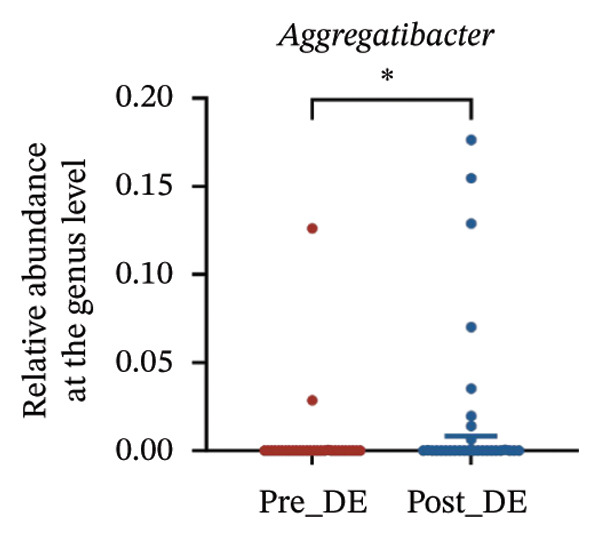
(h)

**Figure:**
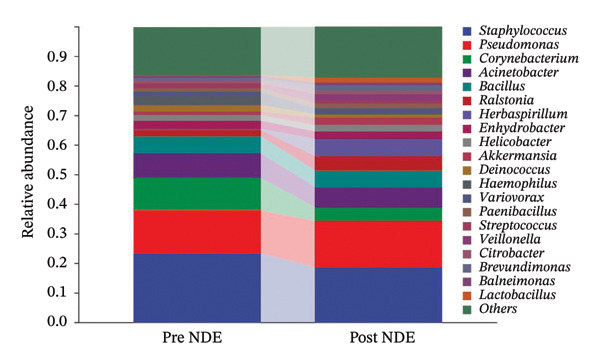
(i)

**Figure:**
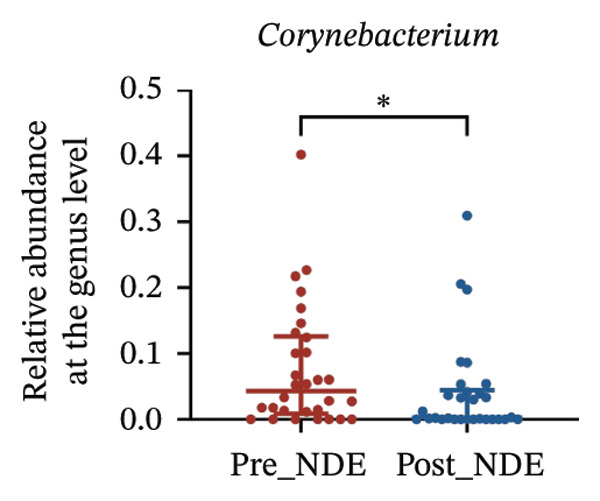
(j)

**Figure:**
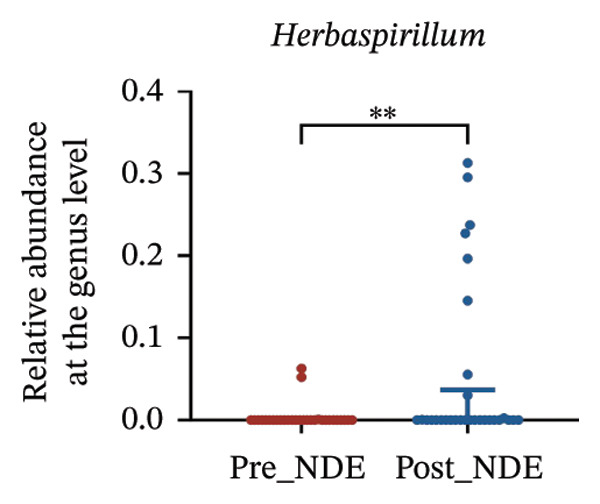
(k)

**Figure:**
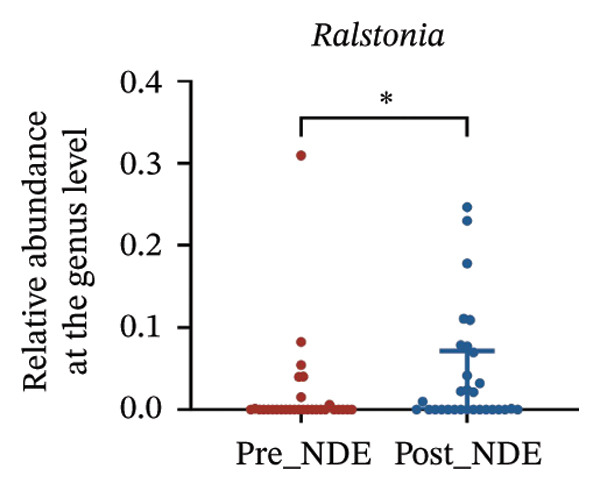
(l)

In NDE patients, phylum‐level stability (*p* > 0.05) was also maintained, but genus‐level changes emerged (Figure 6(i)). Notably, *Corynebacterium* decreased significantly (*p* < 0.01), while *Herbaspirillum* and *Ralstonia* increased postoperatively (*p* < 0.05; Figures 6(j), 6(k), 6(l)). The distinct microbial responses between groups, such as *Pseudomonas* depletion occurring only in DE, suggest that host‐specific factors, including tear composition, epithelial recovery, or baseline neuroimmune status, modulate microbial remodeling after surgery.

## 4. Discussion

FS‐LASIK persists as a cornerstone refractive surgical technique, yet postoperative DE frequently compromises visual outcomes and patient satisfaction. Growing evidence implicates OSM in maintaining corneal homeostasis, with dysbiosis being increasingly associated with corneal pathology [20, 21]. Nevertheless, how baseline microbial profiles interact with FS‐LASIK‐induced alterations to predispose patients to DE remains poorly characterized. Through combined longitudinal and cross‐sectional analyses, this study identified distinct microbial signatures in DE patients, indicating that procedural dysbiosis may contribute to DE pathogenesis after FS‐LASIK.

Baseline clinical data and OSM samples were collected from patients scheduled to undergo FS‐LASIK. During a 3‐month follow‐up, participants were stratified into DE and NDE groups based on established diagnostic criteria. OSM changes were assessed using 16S rRNA sequencing. Preoperative comparisons showed no significant differences in baseline clinical parameters between the two groups, confirming their comparability. Postoperative evaluations, consistent with previous findings, revealed significantly reduced corneal nerve parameters in the DE group, indicating more severe corneal nerve damage [19].

Comparative microbiome analysis identified elevated α‐diversity in DE patients compared to NDE counterparts. Venn diagram quantification detected increased unique OTUs in the DE cohort, denoting enhanced microbial heterogeneity at the ocular surface. This finding is consistent with the results reported by Zhang et al., who observed elevated α‐diversity and OTU counts in diabetic patients with DE, indicating a potential association between ocular surface diseases and microbial diversity [22].

At the phylum level, Proteobacteria, Firmicutes, and Actinobacteria were the predominant phyla across all samples, aligning with previous studies [23]. Postoperatively, the relative abundance of Proteobacteria in the DE group decreased markedly from 41.7% to 34.9%, while Firmicutes increased from 35.1% to 40.8%. Although Proteobacteria are commonly enriched in ocular diseases such as blepharitis and corneal ulcers [24], their reduction in the DE group may reflect a transient dysbiosis of the ocular surface rather than a stable microbial signature. The relative stability of the microbial community in the NDE group further highlights the disrupted microbiota observed in DE patients.

At the genus level, *Streptococcus* was significantly more abundant in the DE group compared to the NDE group, whereas *Pseudomonas* and *Lactobacillus* were significantly reduced, consistent with previous reports of elevated *Streptococcus* in DE patients [25, 26]. Among the identified species, *Streptococcus pneumoniae* is closely associated with mucosal barrier disruption. It secretes a zinc metalloproteinase that cleaves the ectodomain of transmembrane mucin MUC16, impairing its interaction with galectin‐3 on the corneal epithelium [27]. Galectin‐3 serves as a structural bridge between mucins and membrane lipids and is essential for maintaining epithelial barrier integrity [28–30]. Proteolytic cleavage of MUC16, together with goblet cell dysfunction and reduced mucin secretion, may initiate a vicious cycle of microbial overgrowth and barrier compromise. In contrast, *Pseudomonas*, a Gram‐negative bacterium reliant on moist or aqueous environments to maintain biofilm activity [31], was depleted in DE, likely reflecting reduced tear secretion and a desiccated ocular surface. The loss of *Lactobacillus* may result from oxidative stress or interspecies nutrient competition, both detrimental to its survival. The consequent decline in anti‐inflammatory metabolites, such as short‐chain fatty acids, may further favor the expansion of opportunistic pathogens, underscoring the role of dysbiosis in DE pathogenesis [32].

A longitudinal analysis of OSM before and after FS‐LASIK revealed distinct microbial dynamics between DE and NDE patients. In the DE group, postoperative samples showed significant increases in *Staphylococcus*, *Ralstonia*, *Veillonella*, and *Aggregatibacter*, accompanied by notable reductions in *Pseudomonas*, *Corynebacterium*, and *Helicobacter*. As a Gram‐positive bacterium tolerant to desiccation and hyperosmotic environments [33], *Staphylococcus* readily colonizes the dry ocular surface and promotes chronic inflammation via biofilm formation, thereby exacerbating DE symptoms. Among these changes, the significant postoperative increase in *Staphylococcus* in DE patients warrants particular attention. *Staphylococcus* species, particularly *S. aureus*, can disrupt epithelial barrier integrity, stimulate innate immune responses, and create a proinflammatory microenvironment that may hinder corneal nerve regeneration. This dual role—exacerbating ocular surface inflammation and impairing nerve recovery—suggests that *Staphylococcus* overgrowth is not merely a byproduct of surgical stress but may actively contribute to the pathogenesis and persistence of post‐LASIK DE. In contrast, the NDE group exhibited only mild changes, with slight reductions in *Corynebacterium* and modest increases in *Herbaspirillum* and *Ralstonia*, and no significant change in *Staphylococcus* levels. This discrepancy supports the notion that *Staphylococcus*‐driven dysbiosis may be selectively involved in DE progression. Targeting *Staphylococcus*‐associated inflammation could therefore represent a potential therapeutic strategy to enhance ocular surface and nerve recovery following surgery. Notably, both groups showed postoperative decreases in *Corynebacterium* and increases in *Ralstonia*, suggesting that disruption of the stromal nerve plexus during FS‐LASIK may reduce neurotrophic factors, impair epithelial barrier function, and facilitate colonization by opportunistic bacteria [34]. The relatively limited microbial shifts in the NDE group further indicate that pronounced ecological fluctuations may be closely linked to DE development.

Although baseline clinical parameters were comparable between groups, preoperative microbiota composition differed significantly. At the phylum level, the DE group exhibited reduced Cyanobacteria and elevated Verrucomicrobia. At the genus level, *Acinetobacter* abundance was lower, while *Akkermansia* was significantly increased. Cyanobacteria are known producers of antioxidant and anti‐inflammatory metabolites that support epithelial barrier integrity [35]; their depletion may heighten susceptibility to oxidative and inflammatory insults. Conversely, while *Akkermansia* has been shown to degrade gel‐forming mucins and modulate mucosal barrier function in the gut [36], its increased abundance on the ocular surface raises the hypothesis that similar mechanisms might occur in the eye. However, whether *Akkermansia* can degrade ocular mucins such as MUC5AC remains unknown and requires further investigation.

Correlation analysis revealed significant links between specific microbial genera and DE‐related clinical parameters. Elevated *Staphylococcus* abundance correlated with higher OSDI scores and impaired corneal nerve regeneration. Similarly, higher *Aggregatibacter* levels were associated with shorter TBUT, reduced Schirmer I values, and increased OSDI scores, suggesting a role in tear film instability via biofilm formation and chronic inflammation. In contrast, *Lactobacillus* abundance correlated positively with Schirmer I values and CNFD and negatively with OSDI scores, indicating a potential protective role through anti‐inflammatory metabolite production and immune modulation. These findings underscore the complex role of microbial dysbiosis in FS‐LASIK‐associated DE and suggest that microbiota‐targeted interventions may offer new therapeutic avenues. However, an important unresolved question is whether microbiota dysbiosis impairs corneal nerve regeneration, or conversely, whether nerve transection and subsequent microenvironmental disruption caused by FS‐LASIK induce secondary shifts in the microbial community. Corneal nerves regulate tear secretion, epithelial stability, and immune signaling—all of which can influence microbial composition. Therefore, nerve injury itself may confound microbial alterations observed postoperatively. On the other hand, emerging evidence suggests that commensal microbes may actively participate in nerve repair via immunometabolic pathways. Importantly, there were no significant differences in preoperative nerve parameters between the DE and NDE groups, suggesting that the postoperative divergence in nerve outcomes is not attributable to baseline differences. While our findings cannot establish causality, the fact that certain dysbiosis patterns (e.g., elevated *Staphylococcus*, reduced *Lactobacillus*) were predictive of long‐term CNFL integrity, even after adjusting for surgical factors, supports the hypothesis that microbial composition may actively modulate neuroregeneration. These observations point toward a possible additive or synergistic role of surgical trauma and microbiota imbalance in shaping nerve recovery. Future mechanistic studies using gnotobiotic models, co‐culture systems, or early postinjury sampling will be essential to disentangle these bidirectional influences.

Several limitations should be noted. First, although microbial changes were observed at 3 months post‐op, causality and long‐term impact remain unclear. Future studies using metabolomics, co‐culture systems, or gnotobiotic models are warranted to assess functional effects on the tear film and nerve repair. Second, the single‐center design and relatively small sample size may limit statistical power and generalizability. Third, patients were grouped into DE and NDE based on a single postoperative time point, which may not fully capture the heterogeneity or progression of ocular surface changes. Future studies with larger, multicenter cohorts and longitudinal symptom‐based stratification are needed to validate these findings and refine risk prediction models.

In conclusion, this study provides the first evidence that FS‐LASIK is associated with significant alterations in the OSM. Notably, baseline microbial profiles appear to influence postoperative DE susceptibility. Alterations in genera such as *Staphylococcus* and *Lactobacillus* are closely tied to tear film instability and corneal nerve status, underscoring the microbiome’s key role in ocular surface homeostasis. These findings support the value of preoperative microbiome profiling for risk stratification and lay the foundation for personalized prevention and treatment strategies. In addition to their diagnostic relevance, our results suggest that microbial dysbiosis may actively modulate ocular surface recovery after refractive surgery. This highlights the potential for microbiota‐targeted interventions—such as probiotics, bacteriophage therapy, or antimicrobial modulation—to reduce the incidence or severity of postoperative DE. In future studies, we plan to explore the causal role of key microbial taxa using gnotobiotic animal models and assess the therapeutic potential of modulating the OSM to enhance neuroepithelial healing and long‐term visual outcomes.

## Funding

This study was supported by Graduate Innovation Special Fund of Jiangxi Province, YC2024‐B072.

## Ethics Statement

Ethics approval was obtained from the Ethics Committee of the Second Affiliated Hospital of Nanchang University ([2024] No. (34)).

## Conflicts of Interest

The authors declare no conflicts of interest.

## Data Availability

The data that support the findings of this study are available from the corresponding author upon reasonable request.
